# Supplementary material for: Patterns of non-communicable comorbidities at start of tuberculosis treatment in three regions of the Philippines: The St-ATT cohort
Source: PLOS Glob Public Health. 2021 Nov 17;1(11):e0000011. doi: 10.1371/journal.pgph.0000011 (PMC10021424; doi:10.1371/journal.pgph.0000011)
Supplement: S1 File — (DOCX) [file pgph.0000011.s004.docx]

# **Patterns of non-communicable comorbidities at start of tuberculosis treatment in three regions of the Philippines: The St-ATT cohort**

# Supplementary Tables

**Supplementary Table 1. Enrolment by Health facility and TB treatment regimen**

| **Health facility** | **DS-TB** | **MDR-TB** |
| --- | --- | --- |
| San Lazaro Hospital | 99 (12.9) | 16 (12.0) |
| San Nicholas health center | 35 (4.6) | - |
| Pedro Gill health center | 34 (4.4) | - |
| Dr Pablo Torres Memorial Hospital (Riverside) | - | 37 (22.6) |
| La Carlota Hospital | - | 31 (23.3) |
| Bacolod health center | 52 (6.8) | - |
| Bago City health center | 116 (5.1) | - |
| Valladolid rural health unit | 136 (17.7) | - |
| Eversley Hospital | - | 56 (42.1) |
| Carmen rural health unit | 57 (7.4) | - |
| Compostela health center | 82 (10.7) | - |
| Consolacion health center | 99 (12.9) | - |
| Lapu Lapu health center | 50 (6.5) | - |
|  | **767 (100.0)** | **133 (100.0)** |

*Table shows those included in analysis dataset.*

**Supplementary Table 2. Socio-demographic and TB-related characteristics of study participants at enrolment by Region**

| **Characteristic** | | **All (N=900)** | **Manila (N=184)** | **Cebu (N=344)** | **Negros (N=372)** | **p-value** |
| --- | --- | --- | --- | --- | --- | --- |
| **Socio-demographic characteristics** | |  |  |  |  |  |
| Age group at enrolment | 18-40 years old | 380 (42.2) | 90 (48.9) | 165 (48.0) | 125 (33.6) | <0.001 |
|  | 41-65 years old | 419 (46.6) | 84 (45.7) | 147 (42.7) | 188 (50.5) |  |
|  | ≥ 65 years old | 101 (11.2) | 10 (5.4) | 32 (9.3) | 59 (15.9) |  |
| Marital status | Single | 374 (41.6) | 85 (46.2) | 159 (46.2) | 130 (34.9) | <0.001 |
|  | Married | 428 (47.6) | 84 (45.7) | 161 (46.8) | 183 (49.2) |  |
|  | Divorced/separated/widowed | 98 (10.9) | 15 (8.2) | 24 (7.0) | 59 (15.9) |  |
| Highest level of education | Primary | 258 (28.7) | 39 (21.2) | 92 (26.7) | 127 (34.1) | <0.001 |
|  | Secondary | 420 (46.7) | 92 (50.0) | 161 (46.8) | 167 (44.9) |  |
|  | Tertiary or vocational | 214 (23.8) | 53 (28.8) | 84 (24.4) | 76 (20.4) |  |
| Employed | No | 560 (62.2) | 121 (65.8) | 204 (59.3) | 235 (63.2) | 0.297 |
|  | Yes | 339 (37.7) | 63 (34.2) | 140 (40.7) | 136 (36.6) |  |
| Family income | Less than 5,000 PHP | 372 (41.3) | 64 (34.8) | 79 (23.0) | 229 (61.6) | <0.001 |
|  | 5000 - 9999 PHP | 243 (27.0) | 49 (26.6) | 97 (28.2) | 97 (26.1) |  |
|  | 10,000 - 14,999 PHP | 171 (19.0) | 31 (16.8) | 111 (32.3) | 29 (7.8) |  |
|  | 15,000-19,999 PHP | 41 (4.6) | 14 (7.6) | 20 (5.8) | 7 (1.9) |  |
|  | 20,000 PHP or more | 44 (4.9) | 15 (8.2) | 21 (6.1) | 8 (2.2) |  |
|  | Don't know | 28 (3.1) | 11 (6.0) | 16 (4.7) | 1 (0.3) |  |
| Food Insecurity | Food Secure | 675 (75.0) | 117 (63.6) | 279 (81.1) | 279 (75.0) | <0.001 |
|  | Moderate Food Insecurity | 169 (18.8) | 46 (25.0) | 52 (15.1) | 71 (19.1) |  |
|  | Severe Food Insecurity | 56 (6.2) | 21 (11.4) | 13 (3.8) | 22 (5.9) |  |
| Covered by Health Insurance |  | 557 (61.9) | 103 (56.0) | 176 (51.2) | 278 (74.7) | <0.001 |
| Median Household size (IQR) | Adult (18+) | 2 (1-3) | 1 (1-2) | 2 (1-3) | 2 (1-3) | <0.001 |
|  | Young (5-18) | 1 (0-2) | 0 (0-1) | 1 (0-2) | 1 (0-2) | 0.011 |
|  | Children (0-5) | 0 (0-1) | 0 (0-0) | 0 (0-1) | 0 (0-1) | 0.331 |
|  | Whole household | 3 (2-5) | 2 (1-4) | 3 (2-5) | 3 (2-5) | 0.002 |
| Median Household density (IQR) | | 2.0 (1.4) | 2.5 (1.9) | 2.1 (1.3) | 1.7 (1.2) | <0.001 |

| **TB-related characteristics** | |  |  |  |  |  |
| --- | --- | --- | --- | --- | --- | --- |
| New treatment or Relapse | New | 598 (66.4) | 122 (66.3) | 248 (72.1) | 228 (61.3) | 0.009 |
|  | Relapse/Failure/TALF/PTOU | 302 (33.6) | 62 (33.7) | 96 (27.9) | 144 (38.7) |  |
| Basis of diagnosis | Clinical diagnosis | 457 (50.8) | 109 (59.2) | 167 (48.5) | 181 (48.7) | 0.036 |
|  | Bacteriologically confirmed | 443 (49.2) | 75 (40.8) | 177 (51.5) | 191 (51.3) |  |
| DSSM grade | Negative | 258 (28.7) | 53 (28.8) | 100 (29.1) | 105 (28.2) | 0.025 |
|  | 1+ | 65 (7.2) | 6 (3.3) | 21 (6.1) | 38 (10.2) |  |
|  | 2+ | 30 (3.3) | 3 (1.6) | 18 (5.2) | 9 (2.4) |  |
|  | ≥3+ | 60 (6.7) | 3 (1.6) | 21 (6.1) | 22 (5.9) |  |
| Household TB history | ≥ 1 HHC ever diagnosed with TB | 131 (14.6) | 29 (15.8) | 26 (7.6) | 76 (20.4) | <0.001 |
| Current TB symptoms | Cough | 795 (88.3) | 139 (75.5) | 301 (87.5) | 355 (95.4) | <0.001 |
|  | Fatigue | 572 (63.6) | 99 (53.8) | 175 (50.9) | 298 (80.1) | <0.001 |
|  | Fever | 350 (38.9) | 46 (25.0) | 122 (35.5) | 182 (48.9) | <0.001 |
|  | Night sweats | 292 (32.4) | 19 (10.3) | 92 (26.7) | 181 (48.7) | <0.001 |
|  | Reduced appetite | 350 (38.9) | 27 (14.7) | 129 (37.5) | 194 (52.2) | <0.001 |
|  | Chills | 186 (20.7) | 6 (3.3) | 30 (8.7) | 150 (40.3) | <0.001 |
|  | Chest pain | 317 (35.2) | 22 (12.0) | 154 (44.8) | 141 (37.9) | <0.001 |
|  | Weight loss | 522 (58.0) | 44 (23.9) | 172 (50.0) | 306 (82.3) | <0.001 |
|  | Haemoptysis | 275 (30.6) | 23 (12.5) | 86 (25.0) | 166 (44.6) | <0.001 |
|  | Other | 95 (10.6) | 48 (26.1) | 34 (9.9) | 13 (3.5) | <0.001 |
| Median duration (days) symptoms before start of Tx (IQR) | | 48.0 (30.0-77.0) | 44.0 (31.0-73.0) | 39.5 (27.0-63.0) | 57.0 (35.0-90.0) | <0.001 |

**Supplementary Table 3. Previous TB treatment history by region and TB Treatment regimen.**

|  | **Metro Manila** | | **Negros** | | **Cebu** | | **All** | |
| --- | --- | --- | --- | --- | --- | --- | --- | --- |
| **Previous treatment outcome** | DS | MDR | DS | MDR | DS | MDR | DS | MDR |
| New | 119 (97.5) | 3 (2.5) | 215 (94.3) | 13 (5.7) | 231 (93.2) | 17 (6.9) | 565 (94.5) | 33 (5.5) |
| Relapse | 41 (89.1) | 5 (10.9) | 91 (74.0) | 32 (26.0) | 55 (65.5) | 29 (34.5) | 187 (73.9) | 66 (26.01) |
| Treatment after Loss to Follow-Up | 5 (71.4) | 2 (28.6) | 4 (28.6) | 10 (71.4) | 2 (66.7) | 1 (33.3) | 11 (45.8) | 13 (54.2) |
| Treatment after failure | 0 (0) | 1 (100) | 0 (0) | 5 (100) | 0 (0) | 6 (100) | 0 (0) | 12 (100) |
| Previous treatment outcome unknown | 3 (37.5) | 5 (62.5) | 1 (50) | 1 (50) | 0 (0) | 3 (100) | 4 (30.8) | 9 (69.2) |
| Total | 168 (91.3) | 16 (8.7) | 311 (83.6) | 61 (16.4) | 288 (83.7) | 56 (16.3) | 767 (85.2) | 133 (14.8) |

**Supplementary Table 4: Univariable associations with diabetes**

| **Characteristic** | **Level** | **N** | **Diabetic** | **Odd ratio (CI)** | **p-value** |
| --- | --- | --- | --- | --- | --- |
| Sex | Female | 266 | 58 (21.8) | 1 | 0.714 |
|  | Male | 615 | 141 (22.9) | 1.07 (0.75-1.51) |  |
| Age group | 18-40 years old | 372 | 36 (9.7) | 1 | <0.001 |
|  | 41-64 years old | 410 | 134 (32.7) | 4.53 (3.03-6.77) |  |
|  | ≥ 65 years old | 99 | 29 (29.3) | 3.87 (2.22-6.72) |  |
| Total number of people living in the household |  | 881 |  | 1.01 (0.94-1.07) | 0.838 |
| Smoking habit | No smoking experience | 367 | 90 (24.5) | 1 | 0.471 |
|  | Current smoker | 202 | 41 (20.3) | 0.78 (0.52-1.19) |  |
|  | Ex-Smoker | 312 | 68 (21.8) | 0.86 (0.60-1.23) |  |
| smoking exposure | No smoking | 367 | 90 (24.5) | 1 | 0.018 |
|  | tertile 1 <4.9 packyears | 169 | 24 (14.2) | 0.51 (0.31-0.83) |  |
|  | tertile 2, <18.6 packyears | 170 | 38 (22.4) | 0.89 (0.58-1.36) |  |
|  | tertile 3, <125 packyears | 175 | 47 (26.9) | 1.13 (0.75-1.70) |  |
| Alcohol consumption | Daily | 129 | 32 (24.8) | 1.04 (0.67-1.62) |  |
|  | Weekly | 126 | 22 (17.5) | 0.67 (0.41-1.10) |  |
|  | Monthly | 52 | 7 (13.5) | 0.49 (0.22-1.11) |  |
|  | Rarely/Never | 574 | 138 (24.0) | 1 | 0.120 |
| alcohol drinks/yr | none/occassional | 574 | 138 (24.0) | 1 | 0.079 |
|  | min/72 | 75 | 15 (20.0) | 0.79 (0.43-1.44) |  |
|  | 73-364 | 98 | 13 (13.3) | 0.48 (0.26-0.89) |  |
|  | 365/max | 134 | 33 (24.6) | 1.03 (0.67-1.60) |  |
| Final MDR status at enrolment | DS-TB | 749 | 154 (20.6) | 1 | <0.001 |
|  | MDR_TB | 132 | 45 (34.1) | 2.00 (1.34-2.98) |  |
| TB New or (Relapse / Tx after Failure / TALF/ PTOU) | New TB case | 584 | 124 (21.2) | 1 | 0.180 |
|  | Relapse/Failure etc | 297 | 75 (25.3) | 1.25 (0.90-1.74) |  |
| Weight change last 3-6 months | Weight stayed same | 172 | 28 (16.3) | 1 | 0.015 |
|  | Weight increase | 70 | 11 (15.7) | 0.96 (0.45-2.05) |  |
|  | Weight decrease | 639 | 160 (25.0) | 1.72 (1.10-2.67) |  |
| appetite affected food intake past month, compared to normal | Severe decrease in food intake | 26 | 5 (19.2) | 0.84 (0.31-2.30) |  |
|  | Moderate decrease in food intake | 315 | 77 (24.4) | 1.15 (0.81-1.63) |  |
|  | No decrease in food intake | 386 | 85 (22.0) | 1 | 0.765 |
|  | Increase in food intake | 154 | 32 (20.8) | 0.93 (0.59-1.47) |  |
| Food security | Food Secure | 663 | 164 (24.7) | 1 | 0.021 |
|  | Mod. Food Insecurity | 163 | 25 (15.3) | 0.55 (0.35-0.87) |  |
|  | Sev. Food Insecurity | 55 | 10 (18.2) | 0.68 (0.33-1.37) |  |
| Education | Primary School | 260 | 49 (18.8) | 1 | 0.258 |
|  | Secondary School | 413 | 103 (24.9) | 1.43 (0.98-2.10) |  |
|  | Tertiary School | 155 | 37 (23.9) | 1.35 (0.83-2.19) |  |
|  | Vocational | 53 | 10 (18.9) | 1.00 (0.47-2.13) |  |
| Marital status | Single | 366 | 40 (10.9) | 1 | <0.001 |
|  | Married | 420 | 141 (33.6) | 4.12 (2.80-6.06) |  |
|  | Divorced/separated | 25 | 4 (16.0) | 1.55 (0.51-4.75) |  |
|  | Widowed | 70 | 14 (20.0) | 2.04 (1.04-3.99) |  |
| Area | Rural | 189 | 38 (20.1) | 1 | 0.352 |
|  | (Peri)Urban | 692 | 161 (23.3) | 1.20 (0.81-1.79) |  |
| Health facility | San Lazaro | 115 | 28 (24.3) | 1 | 0.072 |
|  | San Nicolas | 35 | 4 (11.4) | 0.40 (0.13-1.23) |  |
|  | Valladolid | 132 | 30 (22.7) | 0.91 (0.51-1.65) |  |
|  | Bago City | 105 | 24 (22.9) | 0.92 (0.49-1.72) |  |
|  | Bacolod | 49 | 12 (24.5) | 1.01 (0.46-2.19) |  |
|  | La Carlota | 30 | 10 (33.3) | 1.55 (0.65-3.71) |  |
|  | Compostela | 82 | 14 (17.1) | 0.64 (0.31-1.31) |  |
|  | Carmen | 57 | 8 (14.0) | 0.51 (0.21-1.20) |  |
|  | Consolacion | 99 | 26 (26.3) | 1.11 (0.60-2.05) |  |
|  | Eversley | 56 | 17 (30.4) | 1.35 (0.67-2.76) |  |
|  | Lapu-Lapu | 50 | 7 (14.0) | 0.51 (0.20-1.25) |  |
|  | Riverside | 37 | 14 (37.8) | 1.89 (0.86-4.16) |  |
|  | Pedro Gil | 34 | 5 (14.7) | 0.54 (0.19-1.52) |  |
| Health insurance | No | 336 | 61 (18.2) | 1 | 0.012 |
|  | Yes | 545 | 138 (25.3) | 1.53 (1.09-2.14) |  |
| BMI high categories | Underweight (<18.5kg/m2) | 382 | 54 (14.1) | 0.44 (0.31-0.63) |  |
|  | Normal (18.5-25kg/m2) | 429 | 117 (27.3) | 1 | <0.001 |
|  | Overweight (25-30kg/m2) | 65 | 26 (40.0) | 1.78 (1.04-3.05) |  |
|  | Obese (>30kg/m2) | 5 | 2 (40.0) | 1.78 (0.29-10.77) |  |
| BMI 3 categories | Underweight | 382 | 54 (14.1) | 0.44 (0.31-0.63) |  |
|  | Normal | 429 | 117 (27.3) | 1 | <0.001 |
|  | Overweight/obese | 70 | 28 (40.0) | 1.78 (1.05-3.00) |  |
| BMI low categories | BMI ≥ 18.5 | 499 | 145 (29.1) | 1 | <0.001 |
|  | BMI ≥17 & <18.5 | 180 | 25 (13.9) | 0.39 (0.25-0.63) |  |
|  | BMI ≥16 & <17 | 94 | 14 (14.9) | 0.43 (0.23-0.78) |  |
|  | BMI <16 | 108 | 15 (13.9) | 0.39 (0.22-0.70) |  |
| Waist to hip ratio >=0.9 (male) or >=0.85 (female) | No | 488 | 61 (12.5) | 1 | <0.001 |
|  | Yes | 393 | 138 (35.1) | 3.79 (2.70-5.32) |  |
| Hypertension study definition | No | 686 | 135 (19.7) | 1 | <0.001 |
|  | Yes | 161 | 57 (35.4) | 2.24 (1.54-3.25) |  |
| Anaemia (<11g/dl) | Normal/Mild | 762 | 173 (22.7) | 1 | 0.835 |
|  | Moderate/Severe <11g/dl | 119 | 26 (21.8) | 0.95 (0.60-1.52) |  |
| HIV status baseline | negative | 293 | 67 (22.9) | 1 | 0.668 |
|  | unknown | 579 | 131 (22.6) | 0.99 (0.71-1.38) |  |
|  | positive | 9 | 1 (11.1) | 0.42 (0.05-3.43) |  |
| Duration TB symptoms before treatment | < 1 month | 286 | 70 (24.5) | 1 | 0.435 |
|  | 1-2 months | 290 | 56 (19.3) | 0.74 (0.50-1.10) |  |
|  | 2-3 months | 154 | 37 (24.0) | 0.98 (0.62-1.54) |  |
|  | > 3 months | 151 | 36 (23.8) | 0.97 (0.61-1.53) |  |

**Supplementary Table 5. Univariable associations with hypertension (Systolic blood pressure ≥140 mmHg or diastolic blood pressure ≥90 mmHg).**

| **Characteristic** | **Level** | **N** | **Hypertensive (%)** | **Odd ratio (CI)** | **p-value** |
| --- | --- | --- | --- | --- | --- |
| Sex | Female | 260 | 55 (21.2) | 1 | 0.289 |
|  | Male | 604 | 109 (18.0) | 0.82 (0.57-1.18) |  |
| Age group | 18-40 years old | 364 | 17 (4.7) | 1 | <0.001 |
|  | 41-64 years old | 404 | 106 (26.2) | 7.26 (4.25-12.40) |  |
|  | ≥ 65 years old | 96 | 41 (42.7) | 15.22 (8.08-28.65) |  |
| Total number of people living in the household |  | 864 |  | 0.98 (0.92-1.06) | 0.649 |
| Smoking habit | No smoking experience | 362 | 66 (18.2) | 1 | 0.063 |
|  | Current smoker | 199 | 29 (14.6) | 0.77 (0.48-1.23) |  |
|  | Ex-Smoker | 303 | 69 (22.8) | 1.32 (0.91-1.93) |  |
| smoking exposure | No smoking | 362 | 66 (18.2) | 1 | 0.010 |
|  | tertile 1 <4.9 packyears | 164 | 20 (12.2) | 0.62 (0.36-1.07) |  |
|  | tertile 2, <18.6 packyears | 168 | 33 (19.6) | 1.10 (0.69-1.74) |  |
|  | tertile 3, <125 packyears | 170 | 45 (26.5) | 1.61 (1.05-2.49) |  |
| Alcohol consumption | Daily | 123 | 25 (20.3) | 1.12 (0.69-1.82) |  |
|  | Weekly | 125 | 26 (20.8) | 1.15 (0.71-1.87) |  |
|  | Monthly | 50 | 8 (16.0) | 0.84 (0.38-1.83) |  |
|  | Rarely/Never | 566 | 105 (18.6) | 1 | 0.856 |
| alcohol drinks/yr | none/occassional | 566 | 105 (18.6) | 1 | 0.918 |
|  | min/72 | 72 | 13 (18.1) | 0.97 (0.51-1.83) |  |
|  | 73-364 | 98 | 21 (21.4) | 1.20 (0.71-2.03) |  |
|  | 365/max | 128 | 25 (19.5) | 1.07 (0.66-1.73) |  |
| Final MDR status at enrolment | DS-TB | 734 | 151 (20.6) | 1 | 0.003 |
|  | MDR_TB | 130 | 13 (10.0) | 0.43 (0.24-0.78) |  |
| TB New or (Relapse / Tx after Failure / TALF/ PTOU) | New TB case | 574 | 109 (19.0) | 1 | 0.993 |
|  | Relapse/Failure etc | 290 | 55 (19.0) | 1.00 (0.70-1.43) |  |
| Weight change last 3-6 months | Weight stayed same | 169 | 43 (25.4) | 1 | 0.050 |
|  | Weight increase | 67 | 14 (20.9) | 0.77 (0.39-1.53) |  |
|  | Weight decrease | 628 | 107 (17.0) | 0.60 (0.40-0.90) |  |
| appetite affected food intake past month, compared to normal | Severe decrease in food intake | 24 | 2 (8.3) | 0.31 (0.07-1.36) |  |
|  | Moderate decrease in food intake | 313 | 43 (13.7) | 0.55 (0.37-0.82) |  |
|  | No decrease in food intake | 378 | 85 (22.5) | 1 | 0.006 |
|  | Increase in food intake | 149 | 34 (22.8) | 1.02 (0.65-1.60) |  |
| Food security | Food Secure | 649 | 123 (19.0) | 1 | 0.198 |
|  | Mod. Food Insecurity | 161 | 35 (21.7) | 1.19 (0.78-1.81) |  |
|  | Sev. Food Insecurity | 54 | 6 (11.1) | 0.53 (0.22-1.28) |  |
| Education | Primary School | 249 | 57 (22.9) | 1 | 0.290 |
|  | Secondary School | 411 | 69 (16.8) | 0.68 (0.46-1.01) |  |
|  | Tertiary School | 153 | 29 (19.0) | 0.79 (0.48-1.30) |  |
|  | Vocational | 51 | 9 (17.6) | 0.72 (0.33-1.57) |  |
| Marital status | Single | 363 | 29 (8.0) | 1 | <0.001 |
|  | Married | 410 | 106 (25.9) | 4.02 (2.59-6.23) |  |
|  | Divorced/separated | 25 | 4 (16.0) | 2.19 (0.71-6.82) |  |
|  | Widowed | 66 | 25 (37.9) | 7.02 (3.76-13.13) |  |
| Area | Rural | 174 | 38 (21.8) | 1 | 0.289 |
|  | (Peri)Urban | 690 | 126 (18.3) | 0.80 (0.53-1.20) |  |
| Health facility | San Lazaro | 115 | 16 (13.9) | 1 | 0.111 |
|  | San Nicolas | 35 | 10 (28.6) | 2.48 (1.00-6.11) |  |
|  | Valladolid | 117 | 28 (23.9) | 1.95 (0.99-3.83) |  |
|  | Bago City | 105 | 20 (19.0) | 1.46 (0.71-2.99) |  |
|  | Bacolod | 49 | 8 (16.3) | 1.21 (0.48-3.04) |  |
|  | La Carlota | 28 | 3 (10.7) | 0.74 (0.20-2.75) |  |
|  | Compostela | 82 | 18 (22.0) | 1.74 (0.83-3.66) |  |
|  | Carmen | 57 | 10 (17.5) | 1.32 (0.56-3.12) |  |
|  | Consolacion | 99 | 21 (21.2) | 1.67 (0.82-3.40) |  |
|  | Eversley | 56 | 7 (12.5) | 0.88 (0.34-2.29) |  |
|  | Lapu-Lapu | 50 | 11 (22.0) | 1.75 (0.74-4.09) |  |
|  | Riverside | 37 | 2 (5.4) | 0.35 (0.08-1.62) |  |
|  | Pedro Gil | 34 | 10 (29.4) | 2.58 (1.04-6.39) |  |
| Health insurance | No | 339 | 44 (13.0) | 1 | <0.001 |
|  | Yes | 525 | 120 (22.9) | 1.99 (1.36-2.90) |  |
| BMI high categories | Underweight (<18.5kg/m2) | 378 | 39 (10.3) | 0.40 (0.27-0.60) |  |
|  | Normal (18.5-25kg/m2) | 419 | 93 (22.2) | 1 | <0.001 |
|  | Overweight (25-30kg/m2) | 62 | 29 (46.8) | 3.08 (1.78-5.34) |  |
|  | Obese (>30kg/m2) | 5 | 3 (60.0) | 5.26 (0.87-31.94) |  |
| BMI 3 categories | Underweight | 378 | 39 (10.3) | 0.40 (0.27-0.60) |  |
|  | Normal | 419 | 93 (22.2) | 1 | <0.001 |
|  | Overweight/obese | 67 | 32 (47.8) | 3.20 (1.88-5.46) |  |
| BMI low categories | BMI ≥ 18.5 | 486 | 125 (25.7) | 1 | ` |
|  | BMI ≥17 & <18.5 | 178 | 23 (12.9) | 0.43 (0.26-0.69) |  |
|  | BMI ≥16 & <17 | 94 | 9 (9.6) | 0.31 (0.15-0.63) |  |
|  | BMI <16 | 106 | 7 (6.6) | 0.20 (0.09-0.45) |  |
| Diabetic according to study definition | No | 655 | 104 (15.9) | 1 | <0.001 |
|  | Yes | 192 | 57 (29.7) | 2.24 (1.54-3.25) |  |
| Anaemia (<11g/dl) | Normal/Mild | 749 | 146 (19.5) | 1 | 0.318 |
|  | Moderate/Severe <11g/dl | 115 | 18 (15.7) | 0.77 (0.45-1.31) |  |
| HIV status baseline | negative | 291 | 41 (14.1) | 1 | . |
|  | unknown | 566 | 123 (21.7) | 1.69 (1.15-2.49) |  |
|  | positive | 7 | 0 (0.0) | 1 | . |
| Duration TB symptoms before treatment | < 1 month | 280 | 49 (17.5) | 1 | 0.834 |
|  | 1-2 months | 288 | 58 (20.1) | 1.19 (0.78-1.81) |  |
|  | 2-3 months | 148 | 27 (18.2) | 1.05 (0.63-1.77) |  |
|  | > 3 months | 148 | 30 (20.3) | 1.20 (0.72-1.99) |  |

**Supplementary Table 6. Univariable associations with moderate/severe anaemia (hemoglobin < 11g/dl)**

| **Characteristic** | **Level** | **N** | **Anaemic (%)** | **Odd ratio (CI)** | **p-value** |
| --- | --- | --- | --- | --- | --- |
| Sex | Female | 274 | 40 (14.6) | 1 | 0.510 |
|  | Male | 625 | 81 (13.0) | 0.87 (0.58-1.31) |  |
| Age group | 18-40 years old | 379 | 40 (10.6) | 1 | 0.055 |
|  | 41-64 years old | 419 | 62 (14.8) | 1.47 (0.96-2.25) |  |
|  | ≥ 65 years old | 101 | 19 (18.8) | 1.96 (1.08-3.57) |  |
| Total number of people living in the household |  | 899 |  | 1.00 (0.93-1.08) | 0.935 |
| Smoking habit | No smoking experience | 375 | 40 (10.7) | 1 | 0.104 |
|  | Current smoker | 204 | 33 (16.2) | 1.62 (0.98-2.66) |  |
|  | Ex-Smoker | 320 | 48 (15.0) | 1.48 (0.94-2.32) |  |
| smoking exposure | No smoking | 375 | 40 (10.7) | 1 | 0.174 |
|  | tertile 1 <4.9 packyears | 173 | 25 (14.5) | 1.41 (0.83-2.42) |  |
|  | tertile 2, <18.6 packyears | 175 | 30 (17.1) | 1.73 (1.04-2.89) |  |
|  | tertile 3, <125 packyears | 176 | 26 (14.8) | 1.45 (0.85-2.47) |  |
| Alcohol consumption | Daily | 130 | 27 (20.8) | 1.77 (1.08-2.88) |  |
|  | Weekly | 129 | 12 (9.3) | 0.69 (0.36-1.31) |  |
|  | Monthly | 52 | 6 (11.5) | 0.88 (0.36-2.13) |  |
|  | Rarely/Never | 588 | 76 (12.9) | 1 | 0.053 |
| alcohol drinks/yr | none/occassional | 588 | 76 (12.9) | 1 | 0.045 |
|  | min/72 | 76 | 8 (10.5) | 0.79 (0.37-1.71) |  |
|  | 73-364 | 100 | 9 (9.0) | 0.67 (0.32-1.38) |  |
|  | 365/max | 135 | 28 (20.7) | 1.76 (1.09-2.85) |  |
| Final MDR status at enrolment | DS-TB | 766 | 104 (13.6) | 1 | 0.803 |
|  | MDR_TB | 133 | 17 (12.8) | 0.93 (0.54-1.62) |  |
| TB New or (Relapse / Tx after Failure / TALF/ PTOU) | New TB case | 597 | 76 (12.7) | 1 | 0.371 |
|  | Relapse/Failure etc | 302 | 45 (14.9) | 1.20 (0.81-1.79) |  |
| Weight change last 3-6 months | Weight stayed same | 173 | 15 (8.7) | 1 | 0.004 |
|  | Weight increase | 72 | 4 (5.6) | 0.62 (0.20-1.94) |  |
|  | Weight decrease | 654 | 102 (15.6) | 1.95 (1.10-3.44) |  |
| appetite affected food intake past month, compared to normal | Severe decrease in food intake | 26 | 9 (34.6) | 4.90 (2.05-11.76) |  |
|  | Moderate decrease in food intake | 324 | 55 (17.0) | 1.89 (1.22-2.95) |  |
|  | No decrease in food intake | 390 | 38 (9.7) | 1 | 0.001 |
|  | Increase in food intake | 159 | 19 (11.9) | 1.26 (0.70-2.26) |  |
| Food security | Food Secure | 674 | 86 (12.8) | 1 | 0.056 |
|  | Mod. Food Insecurity | 169 | 21 (12.4) | 0.97 (0.58-1.62) |  |
|  | Sev. Food Insecurity | 56 | 14 (25.0) | 2.28 (1.19-4.35) |  |
| Education | Primary School | 266 | 50 (18.8) | 1 | 0.007 |
|  | Secondary School | 420 | 53 (12.6) | 0.62 (0.41-0.95) |  |
|  | Tertiary School | 159 | 15 (9.4) | 0.45 (0.24-0.83) |  |
|  | Vocational | 54 | 3 (5.6) | 0.25 (0.08-0.85) |  |
| Marital status | Single | 374 | 45 (12.0) | 1 | 0.577 |
|  | Married | 427 | 62 (14.5) | 1.24 (0.82-1.87) |  |
|  | Divorced/separated | 25 | 5 (20.0) | 1.83 (0.65-5.11) |  |
|  | Widowed | 73 | 9 (12.3) | 1.03 (0.48-2.21) |  |
| Area | Rural | 192 | 37 (19.3) | 1 | 0.010 |
|  | (Peri)Urban | 707 | 84 (11.9) | 0.56 (0.37-0.86) |  |
| Health facility | San Lazaro | 115 | 9 (7.8) | 1 | 0.031 |
|  | San Nicolas | 35 | 3 (8.6) | 1.10 (0.28-4.32) |  |
|  | Valladolid | 135 | 28 (20.7) | 3.08 (1.39-6.84) |  |
|  | Bago City | 116 | 20 (17.2) | 2.45 (1.07-5.65) |  |
|  | Bacolod | 52 | 4 (7.7) | 0.98 (0.29-3.34) |  |
|  | La Carlota | 31 | 4 (12.9) | 1.74 (0.50-6.10) |  |
|  | Compostela | 82 | 6 (7.3) | 0.93 (0.32-2.72) |  |
|  | Carmen | 57 | 9 (15.8) | 2.21 (0.82-5.91) |  |
|  | Consolacion | 99 | 16 (16.2) | 2.27 (0.96-5.40) |  |
|  | Eversley | 56 | 6 (10.7) | 1.41 (0.48-4.19) |  |
|  | Lapu-Lapu | 50 | 3 (6.0) | 0.75 (0.19-2.90) |  |
|  | Riverside | 37 | 5 (13.5) | 1.84 (0.58-5.89) |  |
|  | Pedro Gil | 34 | 8 (23.5) | 3.62 (1.28-10.30) |  |
| Health insurance | No | 342 | 45 (13.2) | 1 | 0.835 |
|  | Yes | 557 | 76 (13.6) | 1.04 (0.70-1.55) |  |
| BMI high categories | Underweight (<18.5kg/m2) | 393 | 78 (19.8) | 2.51 (1.67-3.80) |  |
|  | Normal (18.5-25kg/m2) | 435 | 39 (9.0) | 1 | . |
|  | Overweight (25-30kg/m2) | 66 | 4 (6.1) | 0.66 (0.23-1.90) |  |
|  | Obese (>30kg/m2) | 5 | 0 (0.0) | 1 | . |
| BMI 3 categories | Underweight | 393 | 78 (19.8) | 2.51 (1.67-3.80) |  |
|  | Normal | 435 | 39 (9.0) | 1 | <0.001 |
|  | Overweight/obese | 71 | 4 (5.6) | 0.61 (0.21-1.75) |  |
| BMI low categories | BMI ≥ 18.5 | 506 | 43 (8.5) | 1 | <0.001 |
|  | BMI ≥17 & <18.5 | 183 | 31 (16.9) | 2.20 (1.34-3.61) |  |
|  | BMI ≥16 & <17 | 98 | 24 (24.5) | 3.49 (2.00-6.09) |  |
|  | BMI <16 | 112 | 23 (20.5) | 2.78 (1.60-4.85) |  |
| Diabetic according to study definition | No | 682 | 93 (13.6) | 1 | 0.835 |
|  | Yes | 199 | 26 (13.1) | 0.95 (0.60-1.52) |  |
| Hypertension study definition | No | 700 | 97 (13.9) | 1 | 0.318 |
|  | Yes | 164 | 18 (11.0) | 0.77 (0.45-1.31) |  |
| HIV status baseline | negative | 293 | 29 (9.9) | 1 | 0.033 |
|  | unknown | 597 | 89 (14.9) | 1.59 (1.02-2.49) |  |
|  | positive | 9 | 3 (33.3) | 4.55 (1.08-19.17) |  |
| Duration TB symptoms before treatment | < 1 month | 289 | 38 (13.1) | 1 | 0.449 |
|  | 1-2 months | 296 | 37 (12.5) | 0.94 (0.58-1.53) |  |
|  | 2-3 months | 160 | 19 (11.9) | 0.89 (0.49-1.60) |  |
|  | > 3 months | 154 | 27 (17.5) | 1.40 (0.82-2.40) |  |

**Supplementary Table 7. Univariable associations with moderate/severe malnutrition (BMI<17 kg/m^2^)**

| **Characteristic** | **Level** | **N** | **Malnourished (%)** | **Odd ratio (CI)** | **p-value** |
| --- | --- | --- | --- | --- | --- |
| Sex | Female | 274 | 74 (27.0) | 1 | 0.090 |
|  | Male | 625 | 136 (21.8) | 0.75 (0.54-1.04) |  |
| Age group | 18-40 years old | 379 | 102 (26.9) | 1 | 0.041 |
|  | 41-64 years old | 419 | 82 (19.6) | 0.66 (0.47-0.92) |  |
|  | ≥ 65 years old | 101 | 26 (25.7) | 0.94 (0.57-1.55) |  |
| Total number of people living in the household |  | 899 |  | 1.03 (0.97-1.10) | 0.309 |
| Smoking habit | No smoking experience | 375 | 89 (23.7) | 1 | 0.215 |
|  | Current smoker | 204 | 39 (19.1) | 0.76 (0.50-1.16) |  |
|  | Ex-Smoker | 320 | 82 (25.6) | 1.11 (0.78-1.56) |  |
| smoking exposure | No smoking | 375 | 89 (23.7) | 1 | 0.509 |
|  | tertile 1 <4.9 packyears | 173 | 42 (24.3) | 1.03 (0.68-1.57) |  |
|  | tertile 2, <18.6 packyears | 175 | 45 (25.7) | 1.11 (0.74-1.68) |  |
|  | tertile 3, <125 packyears | 176 | 34 (19.3) | 0.77 (0.49-1.20) |  |
| Alcohol consumption | Daily | 130 | 31 (23.8) | 0.97 (0.62-1.52) |  |
|  | Weekly | 129 | 28 (21.7) | 0.86 (0.55-1.37) |  |
|  | Monthly | 52 | 8 (15.4) | 0.57 (0.26-1.23) |  |
|  | Rarely/Never | 588 | 143 (24.3) | 1 | 0.467 |
| alcohol drinks/yr | none/occassional | 588 | 143 (24.3) | 1 | 0.673 |
|  | min/72 | 76 | 15 (19.7) | 0.77 (0.42-1.39) |  |
|  | 73-364 | 100 | 20 (20.0) | 0.78 (0.46-1.32) |  |
|  | 365/max | 135 | 32 (23.7) | 0.97 (0.62-1.50) |  |
| Final MDR status at enrolment | DS-TB | 766 | 166 (21.7) | 1 | 0.005 |
|  | MDR_TB | 133 | 44 (33.1) | 1.79 (1.20-2.67) |  |
| TB New or (Relapse / Tx after Failure / TALF/ PTOU) | New TB case | 597 | 114 (19.1) | 1 | <0.001 |
|  | Relapse/Failure etc | 302 | 96 (31.8) | 1.97 (1.44-2.71) |  |
| Weight change last 3-6 months | Weight stayed same | 173 | 15 (8.7) | 1 | <0.001 |
|  | Weight increase | 72 | 5 (6.9) | 0.79 (0.27-2.25) |  |
|  | Weight decrease | 654 | 190 (29.1) | 4.31 (2.47-7.52) |  |
| appetite affected food intake past month, compared to normal | Severe decrease in food intake | 26 | 15 (57.7) | 6.02 (2.66-13.66) |  |
|  | Moderate decrease in food intake | 324 | 97 (29.9) | 1.89 (1.33-2.68) |  |
|  | No decrease in food intake | 390 | 72 (18.5) | 1 | <0.001 |
|  | Increase in food intake | 159 | 26 (16.4) | 0.86 (0.53-1.41) |  |
| Food security | Food Secure | 674 | 148 (22.0) | 1 | 0.009 |
|  | Mod. Food Insecurity | 169 | 39 (23.1) | 1.07 (0.71-1.59) |  |
|  | Sev. Food Insecurity | 56 | 23 (41.1) | 2.48 (1.41-4.35) |  |
| Education | Primary School | 266 | 73 (27.4) | 1 | 0.001 |
|  | Secondary School | 420 | 107 (25.5) | 0.90 (0.64-1.28) |  |
|  | Tertiary School | 159 | 24 (15.1) | 0.47 (0.28-0.78) |  |
|  | Vocational | 54 | 6 (11.1) | 0.33 (0.14-0.81) |  |
| Marital status | Single | 374 | 97 (25.9) | 1 | 0.249 |
|  | Married | 427 | 87 (20.4) | 0.73 (0.53-1.02) |  |
|  | Divorced/separated | 25 | 7 (28.0) | 1.11 (0.45-2.74) |  |
|  | Widowed | 73 | 19 (26.0) | 1.00 (0.57-1.78) |  |
| Area | Rural | 192 | 52 (27.1) | 1 | 0.174 |
|  | (Peri)Urban | 707 | 158 (22.3) | 0.77 (0.54-1.12) |  |
| Health facility | San Lazaro | 115 | 33 (28.7) | 1 | 0.002 |
|  | San Nicolas | 35 | 8 (22.9) | 0.74 (0.30-1.79) |  |
|  | Valladolid | 135 | 41 (30.4) | 1.08 (0.63-1.87) |  |
|  | Bago City | 116 | 30 (25.9) | 0.87 (0.49-1.55) |  |
|  | Bacolod | 52 | 12 (23.1) | 0.75 (0.35-1.60) |  |
|  | La Carlota | 31 | 14 (45.2) | 2.05 (0.91-4.62) |  |
|  | Compostela | 82 | 13 (15.9) | 0.47 (0.23-0.96) |  |
|  | Carmen | 57 | 11 (19.3) | 0.59 (0.27-1.29) |  |
|  | Consolacion | 99 | 11 (11.1) | 0.31 (0.15-0.65) |  |
|  | Eversley | 56 | 17 (30.4) | 1.08 (0.54-2.18) |  |
|  | Lapu-Lapu | 50 | 8 (16.0) | 0.47 (0.20-1.12) |  |
|  | Riverside | 37 | 5 (13.5) | 0.39 (0.14-1.08) |  |
|  | Pedro Gil | 34 | 7 (20.6) | 0.64 (0.26-1.62) |  |
| Health insurance | No | 342 | 91 (26.6) | 1 | 0.073 |
|  | Yes | 557 | 119 (21.4) | 0.75 (0.55-1.03) |  |
| Diabetic according to study definition | No | 682 | 173 (25.4) | 1 | <0.001 |
|  | Yes | 199 | 29 (14.6) | 0.50 (0.33-0.77) |  |
| Hypertension study definition | No | 700 | 184 (26.3) | 1 | <0.001 |
|  | Yes | 164 | 16 (9.8) | 0.30 (0.18-0.52) |  |
| Anaemia (<11g/dl) | Normal/Mild | 778 | 163 (21.0) | 1 | <0.001 |
|  | Moderate/Severe <11g/dl | 121 | 47 (38.8) | 2.40 (1.60-3.59) |  |
| HIV status baseline | negative | 293 | 73 (24.9) | 1 | 0.568 |
|  | unknown | 597 | 134 (22.4) | 0.87 (0.63-1.21) |  |
|  | positive | 9 | 3 (33.3) | 1.51 (0.37-6.18) |  |
| Duration TB symptoms before treatment | < 1 month | 289 | 46 (15.9) | 1 | <0.001 |
|  | 1-2 months | 296 | 64 (21.6) | 1.46 (0.96-2.22) |  |
|  | 2-3 months | 160 | 49 (30.6) | 2.33 (1.47-3.70) |  |
|  | > 3 months | 154 | 51 (33.1) | 2.62 (1.65-4.14) |  |
