# Supplementary material for: Patterns of non-communicable comorbidities at start of tuberculosis treatment in three regions of the Philippines: The St-ATT cohort
Source: PLOS Glob Public Health. 2021 Nov 17;1(11):e0000011. doi: 10.1371/journal.pgph.0000011 (PMC10021424; doi:10.1371/journal.pgph.0000011)
Supplement: Attachment — Submitted filename: Comorbidities_PloS Global Health responses 1_.docx [file pgph.0000011.s005.docx]

Responses to reviewers comments: PloS Global Health

**REVIEWER 1:**

Reviewer #1: Review
The authors present important data on the pattern of multi-morbidity among adult patients with both drug susceptible and multi drug resistant tuberculosis in the Philippines. Whilst the subject is very important and there is a lack of published data, there are several key questions and suggestions for improvement that I believe are required prior to consideration for publication.

1. The aim of the study is not explicitly stated. The description of the pattern of comorbidity is not sufficient.

*R1.1: - We have clarified throughout that we report the results of a cross-sectional study here for which the aims were to quantify the prevalence of co-morbidities and identify* *predictors of co-morbidities amongst Filipinos newly starting TB treatment, enrolled in the Starting Anti-TB Treatment (St-ATT) cohort in three regions of the Philippines.*

2. How were patients from treatment centers selected? Whilst the authors state “all” that were enrolled on treatment, there are only 900 from 6,981 (12.9%) total patients. This small percentage opens the study results to selection bias and is a significant limitation. Whether these results are representative of the total patients relies on how included patients may have been different to non-included patients.

*R1.2: Thank you for allowing us to respond to this important issue. In “Participants” in the methods, we have amended the text slightly to now read: “ All non-pregnant adults (≥18 years) registered at participating clinics and within 5 days of starting their TB treatment for pulmonary TB (bacteriologically confirmed or clinically diagnosed) on either standard treatment or on the WHO shorter regimen for multi-drug resistant TB (MDR-TB) were* ***potentially*** *eligible”.*

*Study logistical constraints meant that we could not place research nurses in each clinic location every day. In order to increase representativeness within each area, we elected to increase the number of clinics to recruit from, with fewer days recruiting from each. Study participants were thus approached and enrolled sequentially on the days in which nurses could attend the clinics. As described in lines 180-186, the small percentage of enrolled compared to potentially eligible participants was mostly due to the one very large clinic in the regional capital (Bacolod) of Negros Occidental with a huge catchment area, much of which we ended up not being able to cover due to very unreliable transport and political instability, which we had not fully anticipated in the planning, whilst the number of cases from this clinical site was also much greater than we had observed in our planning phase. However, as can be seen in Figure 1 and in the descriptive text, there were no obvious differences in the age and sex structure of the enrolled vs all registered patients. We acknowledge that there is likely, as in all similar studies, some selection bias. Enrolled participants are likely to be those that may be more likely to complete treatment, as a function of not being resident in the harder to reach barangays and those that were sometimes put forwards for enrolment by the National TB Programme Nurses who would facilitate participants to start treatment on days in which they knew the study nurses would be present.*

*We have amended the discussion under the section strengths and limitations to include the possibility of selection bias and general representativeness of the study population.*

*The clinics outside of Metro Manila were selected to represent the 84 % of urban, peri-urban and rural TB cases occurring outside of the National Capital Region. Region VI contributes 9% of all national registered cases and Region VII, Central Visayas 7%, ranking 3^rd^ and 4^th^ for number of cases. Western Visayas has a case incidence only just below that of NCR (381 vs 384 per 100,000), despite this region being mostly rural. The clinics in Metro Manila were selected to represent those mostly serving the urban poor, including those residing in slum areas.*

3. Why were non pulmonary TB patients excluded from the study?

*R1.3: Non-pulmonary TB cases were excluded to increase the disease homogeneity of the study population and to increase the proportion with bacterial confirmation of disease. In this setting non-pulmonary TB is difficult to diagnose and is often diagnosed very late with very poor outcomes.*

4. The sample size justification is not clear. The authors state that it was calculated by “the primary objective of the cohort”. However, per my first point, this is not clear.

*R1.4: The aim of the cohort study formed the basis of the sample size calculations and therefore provided a fixed sample size for analysis of the cross-sectional data reported here. We have added the aim of the main cohort and the details of the cohort sample size calculation into the text (lines 148-158).*

5. Table 1 (and other results presented) are given by enrolment region. Whilst relevant locally, unless the regions provide the reader with an important message by virtue of their representation and applicability to other settings, I suggest that this is changed. It would be more helpful, for example to present the data as drug susceptible versus multi drug resistant tuberculosis. This would be more applicable to other settings and provide more scope for discussion of the implications of the results.

R1.5: *Thank you - yes, we agree. For Table 1 we provide both data summaries; by region in the main body and by DS vs MDR in the supplementary tables. We have switched these tables so that Table 1 reports the results by TB treatment type (DS- vs MDR-TB). Results shown in figures (including supplementary figures and tables) report the results by both region and TB treatment type and propose to leave unchanged showing both. Table 2 reports the multivariable risk factors for the comorbidities and includes Tb treatment type, whilst the supplementary Tables of the univariable risk factor analyses include both Region and TB treatment type.*

6. What are the implications of these results? The authors do not sufficiently explain why the diagnosis of multi morbidity at TB diagnosis is important and what implications it has for TB programs.

*R1.6: We have amended the first paragraph of the discussion to highlight the importance of meeting the health care needs of persons with TB and comorbidities at the start of treatment as per the WHO End TB strategy, both for the potential of directly or indirectly improving TB treatment outcomes, for health equity and to protect other at-risk clinic populations, e.g. diabetics from being exposed to those with potentially infectious TB. A subsequent report will summarise whether there is evidence of an association between comorbidities and adverse TB treatment outcomes.*

7. For example, they demonstrate that 68% of patients have poorly controlled diabetes mellitus (HbA1c >8%). It may be possible therefore, that better glycaemic control through x intervention at TB diagnosis results in better treatment outcomes or less post TB sequelae.

*R1.7: Yes, this is certainly biologically plausible and the rationale for the WHO recommendations and this study. Data are limited so far, especially in LMIC settings of the effects of multi-comorbidities on TB treatment and post treatment outcomes, or how best to implement interventions. As noted above, evidence from this study will form the basis of another manuscript.*

8. Are TB treatment outcomes known for this cohort? It would be incredibly important to know if the outcomes were worse among specific co-morbidities.

*R1.8 – data cleaning and analysis are underway and will form a follow-on manuscript*

**REVIEWER 2:**

Introduction
1. The paper is generally well-written. However, I suggest that the paragraph on Page 4 be divided into 2 paragraphs, at least. A single paragraph should cover one page.

*R2.1. Done.*

2. Please write the acronyms for DS-TB and MDR-TB in full when first used.

*R2.2. Done.*

Methods
3. Study design: While the overall study may be prospective, this paper presents cross-sectional data making this a cross-sectional study. Please amend accordingly.

*R2.3. Done.*

4. Line 78: Please write the acronym DOT in full.

*R2.4. Done.*

5. Setting: What were the criteria for the selection of the TB-DOTS clinics i.e. why were these specific clinics selected for inclusion in this study?

*Please see our response to a related comment – 1.2 above. In addition to these criteria, there were also pragmatic criteria of recommendations of the local TB control programme staff and logistics of convenient laboratory capacities and space for research staff.*

6. Overweight/obesity, underweight, and raised waist-to-hip ratio are all mentioned in the Results section and need to be defined in the Methods, please.

*R2.6. Done.*

7. Please include a definition of food insecurity.

*R2.7. Done. We have added another reference to the online tool and described the categorisation based on the raw scores.*

8. Lines 118-120: Reference is made to hip circumference measurement, but this is not described.

*R2.8. We have added a sentence for hip circumference to the anthropometry meethods and have also added the definition of the waist-to-hip ratio (please see R2.6).*

9. Line 131” ‘calculate the’ should come before ‘number’

*R2.9. Grammar corrected.*

10. Data collection: How did fieldworkers enrol participants i.e. select from the potentially eligible participants? For example, using random tables, etc.

*R2.10. Please see above response to a related comment R1.2*

Results
11. The study dates (1st August 2018 and 21st February 2020) belong in the Methods, please.

*R2.11. Done.*

Table 1:
12. For your international reader, please write PHP in full in the footnote and the equivalent of 1 PHP to the USD.

*R2.12. Done.*

13. Please write the following acronyms in full: TALF/PTOU

*R2.13. Thank you, we have added this to the footnotes of the table where we use this abbreviation.*

14. Line 236: Why was a cut-point of 8% and not 7% used for diabetes control? Please include the appropriate international/standard/WHO reference.

*R2.14. We have followed the pragmatic cut-point of 8% for persons with TB-DM recommended by the working group of the Union against Tuberculosis and Lung Disease, which is currently the most authoritative guideline for the management of diabetes in persons with TB. We have inserted a reference to this document earlier in the manuscripts as well as the discussion.*

15. Lines 279-280: ‘were considered’ is repeated.

*R2.15. Done*

16. Please be consistent with the use of either blood pressure or BP in the text – do not use both.

*R2.16. Done.*

17. Line 313: ‘all grades of anaemia’ – please provide the haemoglobin cut-point and the relevant reference

*R2.17. Done*

Discussion
18. Lines 411-414 need rephrasing for better clarity, please. I suggest splitting into 2 sentences for easier understanding.

*R2.18. Done.*

19. Lines 407-432: This paragraph is difficult to follow. I suggest dividing into 3-4 paragraphs with shorter sentences for ease of reading.

*R2.19 WE apologise for the lack of clarity and have amended the text and hope it is now more explicit and separated into more paragraphs and separate sentences.*

20. Lines 440-441: Please amend and remove the emphasis of including systolic blood pressure of 120-129 mmHg as being potentially classified as hypertension.

*R2.20. The text has been amended.*

21. Lines 446-450; 458-462, 493-497: Please divide into at least 2 sentences each.

*R2.21. Done*

22. Line 469: Please include the reference.

*R2.22. Done*

23. Line 473: Please remove ‘prospective’ – this data is from baseline analyses of a prospective study i.e. cross-sectional assessments were done in this study.

*R2.23. Done*
